# Mental health and catastrophic health expenditures in conflict-affected regions of Colombia before and during COVID-19: an inequalities perspective

**DOI:** 10.1186/s12939-025-02485-4

**Published:** 2025-05-22

**Authors:** Sebastian Leon-Giraldo, Nicolas Jater-Maldonado, Javier Garcia-Estevez, Oscar Bernal

**Affiliations:** 1 School of Government, Universidad de los Andes, Bogotá, Colombia; 2 The Interdisciplinary Centre of Development Studies, Universidad de los Andes, Bogotá, Colombia

## Abstract

The objective of this study is to analyze the changes in catastrophic health expenditures (CHE) and out-of-pocket expenditures (OOP) before and during the COVID-19 pandemic, as well as to examine their determinants in Meta, Colombia, a region affected by armed conflict. We used data from the Conflicto, Paz y Salud (CONPAS) survey and applied mixed-effects logistic regression models. The analysis places particular emphasis on mental health as a key determinant, comparing the odds of incurring OOP and CHE between individuals with and without a tendency to present mental health disorders (SRQ + versus SRQ-).

The results show that the odds of incurring CHE increased in 2020 compared to 2018, while the odds of incurring OOP decreased during the same period. Individuals living in less wealthy households (quintiles 1, 2, and 3 of the Household Wealth Index) have more odds of incurring CHE than those in the wealthiest group (quintile 5). Similarly, individuals aged 45 to 60 years or over 60 years and have more odds of incurring CHE than younger individuals (18 to 44 years). Those who fell sick or were hospitalized also have more odds of incurring CHE compared to those who did not. Additionally, we found that individuals with SRQ + have significantly higher odds of incurring OOP and marginally significantly higher odds of incurring CHE compared to SRQ- individuals. Additionally, those who have been displaced due to the conflict have higher odds of incurring OOP compared to those who have not.

This study underscores the heightened vulnerability of regions impacted by violence; a situation further exacerbated by the COVID-19 pandemic. It emphasizes the need for targeted financial safeguards and comprehensive mental health programs to support marginalized communities, enhance economic resilience, and advance progress toward the Sustainable Development Goals (SDGs), particularly SDG 3, which aims to promote good health and well-being. The findings shed light on health disparities in violence-affected areas, highlighting the urgency of policies designed to improve financial security and healthcare access for individuals with mental health conditions.

**Supplementary Information:**

The online version contains supplementary material available at 10.1186/s12939-025-02485-4.

## Introduction

The prolonged armed conflict in Colombia has greatly influenced its development path, impacting millions of people through fatalities, forced migration, and extensive socio-economic instability. From 1958 to 2022, the conflict led to greater than 262,000 fatalities, 80,000 cases of forced disappearances, and approximately 8.4 million individuals internally displaced [1]. The conflict’s consequences go beyond the current violence, causing significant disruption to healthcare infrastructure and economic stability [2], resulting in limited availability of medical supplies and a shortage of skilled healthcare professionals [3]. Additionally, it has hampered households’ ability to generate income, thereby undermining their economic well-being [4]. The limitation of accessible and free healthcare services in conflicting contexts can increase financial difficulties [5, 6].

Research has shown an elevated incidence of mental health conditions, including depression, anxiety, and post-traumatic stress disorder, among those who have experienced extended violence and displacement [7, 8]. The mental health difficulties are further complicated by socio-economic difficulties, resulting in an intricate situation that obstructs progress and growth [9, 10]. Some studies examined mental health inequalities before and after a peace agreement and found considerable differences in regions affected by violence, highlighting the necessity for specific mental health interventions [11, 12]. While there has been of some research conducted on catastrophic health expenditures, including in conflict affected territories [13–15], further research is needed to explore the relationship between OOP expenditures in LMIC and non-communicable diseases (NCD), including mental health disorders [16]. Our study contributes to literature by exploring the determinants of OOP and CHE in areas affected by armed conflict, with an emphasis on mental health as a determinant.

Socio-economic factors prevalent mainly in LMICs such as poverty, income inequality, inadequate access to healthcare services, violence including civil conflicts, crimes, domestic violence, income inequality, unemployment, and lack of access to clean water, sanitation, and other basic services have a significant impact on mental health issues [17–20]. In addition, there exists a reciprocal correlation between mental health and poverty, as mental health disorders can result in economic hardships due to diminished capacities, while mental health problems can also result from poverty due to stress, lack of access to resources, and social stigma associated with living in poverty, thereby sustaining a cycle of poverty [21]. The combination of these elements, together with extended violence, in Low- and Middle-Income Countries (LMICs) such as Colombia, provides a complicated setting for mental health [20, 22, 23]. All this situation was exacerbated by the adverse impacts of the COVID-19 pandemic [24].

In addition to these economic and social factors present in LMIC, these countries also face challenges in providing the necessary infrastructure and resources, both financial and human, to adequately address mental health, making the situation more challenging than in high-income countries (HIC) [18, 25]. These differences mentioned between LMICs and HICs could also be present between middle-income and low-income countries, however, most literature distinguishes between LMIC and HIC rather than between lower-income and middle-income countries [20, 21, 26–31]. Therefore, we consider Colombia as LMIC based on the World Bank Group country classification [32].

Inequality in health encompasses more than individual differences; it reflects structural and social determinants that systematically disadvantage certain populations over others [10, 33]. These determinants, such as income, education, occupation, and geographical location, shape health outcomes by influencing access to resources, quality of healthcare, and exposure to health risks [34]. According to the World Health Organization (WHO), social determinants of health are “the conditions in which people are born, grow, live, work, and age” and there are avoidable differences in health status observed within and between countries [35].

In conflict-affected Regions like Meta in Colombia, these inequalities are further exacerbated by systemic issues such as infrastructure deficiencies, economic instability, and ongoing violence, all which limit access to healthcare and worsen health outcomes [21, 36]. Mental health disparities in these regions exemplify the compounded effects of social determinants, where marginalized groups face greater barriers to adequate care and a higher risk of CHE [20, 37, 38]. This framework highlights the importance of examining health inequality as a consequence of intersecting social, economic, and structural factors, rather than isolated individual attributes. The focus on mental health arises from the need to analyze its impact on individuals’ economic vulnerability and access to healthcare [17, 18, 20], making it a critical determinant of OOP and CHE in conflict-affected settings [39–41].

The pandemic has underscored the need to integrate mental health into development agendas, elevating it from an often-overlooked issue to a global priority [42]. This shift is emphasized by the Sustainable Development Goals (SDGs), particularly SDG-3, which focuses on good health and well-being [43]. Mental health is often studied through health outcomes, but examining CHE alongside mental health provides valuable insights into access to healthcare services. This approach offers a fresh perspective on understanding mental health in conflict-affected territories.

CHE refer to healthcare costs that exceed a specific percentage of a household’s income, usually 10%, 20%, or 25% of total consumption or income [44]. We apply a 20% threshold to ensure comparability with previous national studies on CHE conducted in Colombia [13, 39, 45]. CHE have a notable impact on household finances by reducing savings, requiring the sale of assets, and encouraging more borrowing, this ultimately results in long-term financial instability and poverty [46]. The financial impact of CHE can discourage individuals from accessing essential medical treatment, leading to worse health results and impeding overall progress [27, 44, 47, 48].

The 2016 Peace Agreement with *Fuerzas Armadas Revolucionarias de Colombia* (FARC-EP), which was the main guerrilla group in the country, represented a promising shift for Colombia, aiming to reduce violence and promote sustainable peacebuilding by addressing five decades of armed conflict that caused profound social, economic, and institutional disruptions [49, 50]. Nevertheless, ongoing difficulties persist, particularly in regions still under the influence of other armed groups or dissidents, who are former FARC-EP members that refused to accept the peace process [51]. These conditions not only worsen health outcomes but also raise the probability of incurring large healthcare expenses, particularly when the health system does not sufficiently cover the costs [11, 13]. OOP expenses frequently result in CHE, which force households into poverty and discourage individuals from getting essential medical treatment [44, 48, 52].

Besides the aforementioned challenges the onset of the COVID-19 pandemic in March 2020 may have worsened these existing vulnerabilities in Colombia [53–55]. The pandemic pushed stress on healthcare systems, escalated unemployment rates, and exacerbated economic conditions, resulting in elevated expenses for healthcare. These expenses involved costs related to testing, treatment, and preventative measures [24, 56, 57]. The combination of these difficulties, along with the loss of employment, ongoing poverty, and mental stress caused by the pandemic, could have lasting effects on healthcare costs and general welfare [24, 53, 54, 58, 59].

This study provides a more comprehensive understanding of the challenges faced by individuals with tendency to present mental health disorders in conflict-affected regions, as it considers the socioeconomic, geographic, and demographic determinants such as income, education, gender, ethnicity, or region and their inequalities within the population. This approach can reveal deeper insights into access to healthcare services and the economic impact of CHE. Furthermore, addressing mental health and CHE within the framework of the Sustainable Development Goals (SDGs) is crucial for promoting Good Health and Well-Being (SDG-3) and achieving sustainable development in conflict-affected regions.

On the other hand, it is important to provide context on the economic conditions of Colombia and the Meta region, therefore, we analyze monetary poverty based on data from the National Administrative Department of Statistics of Colombia (DANE). The incidence of national monetary poverty increased from 34.7% in 2018 to 42.5% in 2020, and the incidence of extreme poverty rose from 8.2% in 2018 to 15.1% in 2020. The Gini coefficient increased from 0.517 in 2018 to 0.544 in 2020. In 2020, in the Meta region, the incidence of monetary poverty was 40%, and extreme poverty was 15.4%. Although overall poverty in the Meta region was lower than the national average, extreme poverty exceeded this average. Regarding inequities, the incidence of poverty in 2020 for women was 46.7%, compared to 40.1% for men. Individuals without education or with only primary education (49.6%) or secondary education (46.3%) have a higher incidence of poverty compared to those with a technical/technological degree (30.2%) or a university degree (15.7%) [60].

At the end of 2020, the unemployment rate in Colombia was 13.8%, higher for women at 18.6% compared to men at 11%. Additionally, the proportion of the population employed informally reached 49%. The unemployment rate in the capital city of the Meta region was 18.6%, and informality reached 59.6%, well above the national average [61]. The incidence of poverty among the unemployed is 69.1%, while among the employed it is 38.7%. Regarding occupational status, the incidence of poverty among salaried workers is 24.7%, while for self-employed individuals it is 50.9%. The household profile is also relevant: the incidence of poverty in a household without children is 27.8%, in a household with one child is 44.7%, and in a household with three or more children is 76.9%. Additionally, a household with four or more residents has a poverty incidence of 50.8% [60].

Furthermore, it is important to briefly explain the Colombian health system: The system is organized into three main affiliation schemes. The first is the contributory regime, which is mandatory for all individuals (contributors and beneficiaries) linked through employment contracts, public servants not legally exempt, and self-employed or informal workers who join voluntarily. The second is the subsidized regime (RS), which includes individuals without the ability to pay. The third is the special regime, which covers workers and members of the Military Forces, the National Police, Ecopetrol (National Oil Company), the teaching profession, and public universities. Additionally, non-affiliated individuals are provided care, when necessary, financed by territorial entities that have mechanisms in place for this purpose [62, 63].

Affiliation to the General System of Social Security in Health (SGSSS) is mandatory and is done through public or private health promotion entities (EPS), which are responsible for offering, at a minimum, the Mandatory Health Plan (POS). Healthcare providers are service provider institutions (IPS), which may or may not be integrated with the EPS but are always contracted by them [63]. According to figures from the Ministry of Health and Social Protection, in 2022, health insurance coverage reached 99.12% of the population enrolled in the system, approximately 45.8% are affiliated with the contributory regime, 49.9% with the subsidized regime, and 4.3% with the special regime. In the Meta region (our study region), coverage reached 97.21% in 2022 [64]. Coverage in Colombia extends to almost the entire population. Moreover, other studies show that the share of OOP expenses in total sector spending is below the global average, making it one of the Latin American countries with the lowest OOP expenses [65]. Additionally, the promotion and the prevention of mental disorders, as well as, comprehensive mental health care, are part of the SGSSS, however, there are challenges in implementing most of the mental health services proposed by Colombian legislation [66].

This study aims to analyze inequalities in CHE in a conflict-affected region of Colombia (Meta), comparing the periods before and during the COVID-19 pandemic and emphasizing mental health as a determinant. The Meta region, located in the central part of Colombia, has been significantly impacted by the armed conflict and represents a critical area for examining the intersection of conflict, health expenditures, and the additional burden imposed by the COVID-19 pandemic. Using data from the Conflicto, Paz y Salud (CONPAS) survey and employing descriptive analyses, as well as mixed-effects logistic models, this research examines the CHE and financial sources used by individuals with and without the tendency to present mental health disorders (SRQ + vs. SRQ).

## Methods

### Data source

We conducted the Conflict, Peace, and Health (CONPAS) survey in 1309 households across the Meta region, one of the areas most affected by the armed conflict in Colombia. The primary goal was to gather data on socioeconomic conditions and health indicators to support public health analyses in the context of post-conflict recovery following the 2016 peace agreement. The initial survey was carried out in 2018, followed by subsequent rounds in 2019 and 2020, targeting the same adult respondents. The 2019 round was answered by 1106 households, and the 2020 round was answered by 905 households from the 1309 initially surveyed in 2018^1^. Although there was a reduction in the sample size, we conducted an attrition analysis^2^ and concluded that there is no evidence of selective data loss.

The 2018 and 2019 interviews were conducted in person between November and December, while the 2020 survey was conducted by telephone from November 2020 to January 2021 due to quarantine and isolation measures during the COVID-19 pandemic. Despite these constraints, the lack of in-person interviews in 2020 did not significantly affect the response rate. In the 2019 survey, 99.01% of participants (1095 out of 1106) reported having access to a cell phone at home, mitigating concerns about telephone survey accessibility.

The CONPAS sample was designed using a probabilistic, stratified approach to ensure representativeness for the total population of Meta, as well as for urban and rural populations. It also accounted for varying levels of conflict exposure, classifying municipalities as “heavily,” “lightly,” or “not affected” by violence based on CERAC’s classification of the historical intensity and persistence of conflict [67]. Additional stratification was included for the regional capital, Villavicencio. Households were selected through multistage sampling, with units chosen via simple random sampling without replacement.

### Outcomes and independent variables

- Health expenditures: Measures how much the household spent on medicines, health supplies (orthopedic shoes, splints, etc.), and health-related services, including the cost of medical services.
- Out of pocket (OOP): It is a binary variable that considers whether the person incurred any out-of-pocket health expenditures.
- Catastrophic health expenditures (CHE): It is a binary variable that considers whether healthcare expenses exceed 20% of the household’s total expenses.

Among the independent variables, the binary SRQ variable is included, which measures the tendency to experience mental health disorders and is calculated based on the Self-Report Questionnaire (SRQ-20), developed by the World Health Organization (WHO) [68]. The questionnaire consists of 20 questions about general mental health and well-being, and an affirmative answer to 8 or more of the 20 questions indicates a positive tendency to experience mental health disorders, specifically Common Mental Disorders (CMD) such as depression and anxiety [68, 69]. It is a globally accepted, practical, and well-validated instrument for measuring individual tendencies towards mental health disorders, specifically CMD [70]. The National Mental Health Survey conducted by the Ministry of Health and Social Protection of Colombia also considers that an affirmative response to 8 or more of the 20 questions on the SRQ-20 indicates a positive tendency towards experiencing a mental health disorder (SRQ+) [69, 71]. While there is strong evidence showing that SRQ is a universally used, reliable, and well-validated instrument, we conducted a statistical test to assess reliability, for which we calculated Cronbach’s alpha^3^.

The socioeconomic variables^4^ encompass the Household Wealth Index (HWI^5^), which measures socio-economic status across five quintiles, with the 5th representing the wealthiest. Additional variables include age group, ethnicity, gender, marital status, educational level, employment status (formal employees, informal employees, and those out of the labor force), and area of residence (rural or urban). Health insurance is classified according to the Colombian SGSSS system into contributory, subsidized, exception, and non-affiliated. Health status indicates whether the person has been sick or hospitalized in the past year. Household size reflects the number of household members, while children under six years old indicates their presence in the household. Displacement due to armed conflict records whether the person has ever been displaced. Finally, the level of conflict impact in the area categorizes regions as no conflict, regional capital city, heavily affected, or lightly affected.

In the first instance, we conducted a descriptive analysis of average health expenditures, expressed in 2020 dollars, for the years 2018, 2019, and 2020. In addition, we generated a comparative table of average health spending between those with any mental health disorder (SRQ+), and those without this diagnosis (SRQ-). This approach allows an initial understanding of the differences in health spending between the two groups. This analysis was carried out considering OOP and CHE.

In the second instance, we estimated two mixed-effects logistic regression models that account for the panel nature of the data by incorporating both fixed parameters and random effects. The use of the mixed-effects logistic regression models was selected in this study based on a combination of statistical tests and model fit criteria^6^. First, the intraclass correlation coefficient (ICC) indicated the presence of unobserved heterogeneity across individuals, justifying the inclusion of random effects to account for this variation. Additionally, the Hausman tests results supported the use of a random-effects or mixed-effects logistic model over a fixed-effects logistic model, as the null hypothesis that unobserved individual effects are uncorrelated with the independent variables could not be rejected. While the Akaike Information Criterion (AIC) and Bayesian Information Criterion (BIC) values for the mixed-effects and random-effects logistic models were comparable, the mixed-effects models were selected based on their ability to account for unobserved individual-level heterogeneity and provide a better fit for the data according to the likelihood-ratio test results. The models were estimated using the maximum likelihood estimator (MLE), which ensures consistent and efficient estimates under the mixed-effects framework [72–74].

Additionally, we performed an attrition analysis which concluded that there was no selective loss of data, that is, there was no bias due to the decrease in the sample over time^7^. The observations correspond to data from the years 2018, 2019 and 2020. The dependent variables considered in the models were:

1. Model 1: Binary variable of incurring or not incurring catastrophic health expenditures (CHE).
2. Model 2: Binary variable of incurring or not incurring out-of-pocket health expenses (OOP).

Both models include as independent variables a series of socioeconomic factors, among others. In this way, the aim is to determine whether the presence of a mental health disorder is associated with higher health expenditures and a greater odd of facing CHE. We used robust standard errors in all estimations.

At the end, we conducted a statistical analysis to identify the main sources of healthcare financing, using separate binary logistic regression models. This methodology allowed us to explore the likelihood of individuals resorting to savings, loans, asset sales, or income, considering the presence of mental health disorders (SRQ+) [73, 75]. The selected models were evaluated using the Hosmer-Lemeshow test and the area under the ROC curve (AUC)^8^. However, it is clarified that these models were developed only with an explanatory approach, aimed at identifying significant relationships rather than maximizing predictive accuracy. This approach ensures more truthful results that are aligned with the nature of the phenomenon studied.

## Results

Table 1 presents the OOP health expenditures for the years 2018, 2019, and 2020. The data is adjusted to constant prices of the year 2020 in USD to account for inflation and provide a clear comparison across years.

**Table 1:** OOP expenditures CONPAS 2018, 2019, 2020 – (Constant prices, year 2020 – USD)

Year	2018 (*N* = 1309)	2019 (*N* = 1106)	2020 (*N* = 905)
Obs.	*1309*	*1106*	*905*
Mean (USD)	*26.84*	*23.13*	*20.04*
Std. Dev	*59.37*	*40.57*	*38.27*
Min	*0*	*0*	*0*
Max	*571.19*	*275.14*	*270.76*

Source: Prepared by authors based on CONPAS 2018, 2019 and 2020

Table 2 provides a detailed comparison of OOP health expenditures based on the presence of mental health disorders (SRQ + vs. SRQ-) for the years 2018, 2019, and 2020.

**Table 2:** OOP expenditures CONPAS 2018, 2019, 2020. According to mental health disorders (SRQ) – (Constant prices, year 2020 - USD)

Year	2018 (*N* = 1309)		2019 (*N* = 1106)		2020 (*N* = 905)	
SRQ	*Positive*	*Negative*	*Positive*	*Negative*	*Positive*	*Negative*
Obs.	*424 (32.39%)*	*885 (67.60%)*	*315 (28.48%)*	*791 (71.51%)*	*223 (24.64%)*	*682 (75.39%)*
Mean (USD)	37.39	21.78	31.16	19.93	27.35	17.64
Std. Dev	70.23	52.69	46.42	37.54	43.65	36.06
Min	0.00	0.00	0.00	0.00	0.00	0.00
Max	571.19	571.19	275.14	275.14	270.76	270.76

Source: Prepared by authors based on CONPAS 2018, 2019 and 2020

These data presented in Table 1 indicate that the overall mean health expenditures decreased each year from 2018 to 2020. Table 2 indicates that people with a positive tendency to present mental health disorders continuously experienced greater medical expenses in comparison to those without such disorders during all three years. The disparity in healthcare spending between people with SRQ + and SRQ- persisted, highlighting the enduring economic strain on those with mental health conditions.

The data reveals important findings concerning healthcare expenses in conflict-affected territories of Colombia throughout the three-year period under investigation. There was an overall decline in the average of OOP expenditures for the entire population from 2018 to 2020. Furthermore, individuals with the tendency of having mental health disorders (SRQ+) consistently had higher OOP health expenditures compared to those without such disorders (SRQ-). The average OOP for those with SRQ + in 2018 was approximately twice as high as that of individuals with SRQ-. Despite a small reduction in 2019 and 2020, those with SRQ + still experienced significantly higher OOP. These findings emphasize the extra stress experienced by those with mental health disorders, highlighting the ongoing disparities in medical expenses.

Overall, these results underscore the importance of targeted financial protection mechanisms to support individuals with mental health disorders and mitigate the economic impact of health expenditures. Addressing these financial burdens is crucial for promoting mental health and overall well-being, particularly in conflict-affected regions where vulnerabilities are compounded by socio-economic and health challenges.

Table 3 below presents data on the number and percentage of individuals who incurred CHE, defined as health expenses exceeding 20% of household consumption, across the years 2018, 2019, and 2020.

**Table 3:** CHE by year 2018, 2019, 2020

Year	2018 (*N* = 1309)		2019 (*N* = 1106)		2020 (*N* = 905)	
Incurred a CHE	***Yes***	***No***	***Yes***	***No***	***Yes***	***No***
*CHE*	*211* *(16.12%)*	*1*,*098* *(83.88%)*	*217* *(19.62%)*	*889* *(80.38%)*	*163* *(18.01%)*	*742* *(81.99%)*

Source: Prepared by authors based on CONPAS 2018, 2019 and 2020

The data shows a slight rise in the proportion of people experiencing catastrophic health expenses in 2019 compared to 2018, followed by a minor decline in 2020. Although, there were shifts from year to year, a considerable number of individuals continued to experience such expenses, highlighting the financial strain that medical expenses place on households. The proportion of individuals experiencing CHE remained largely constant, suggesting a consistent and enduring financial burden throughout the years under investigation.

Table 3 highlights several key points regarding CHE over the three years. Firstly, a notable percentage of individuals incurred catastrophic health expenditures each year, underscoring the important financial burden of health costs on households in conflict-affected regions of Colombia. Despite slight year-to-year variations, the percentage of individuals incurring CHE remained relatively high, indicating ongoing financial challenges. The data from 2020, during the COVID-19 pandemic, shows a slight decrease in the percentage of individuals incurring catastrophic health expenditures compared to 2019. However, the financial burden remained important, reflecting the compounded impact of the pandemic on already vulnerable populations. These findings emphasize the need for targeted interventions to address the financial vulnerabilities associated with health expenditures, particularly in conflict-affected regions. Strategies to reduce OOP expenses and provide adequate financial protection for households are crucial for promoting overall well-being and economic stability.

Table 4 provides a detailed comparison of catastrophic health expenditures between individuals with positive SRQ and negative SRQ in the years 2018, 2019, and 2020.

**Table 4:** CHE a comparison between people with SRQ + vs. SRQ-

Year	2018 (*N* = 1309)		2019 (*N* = 1106)		2020 (*N* = 905)	
SRQ	***Positive*** ***424***	***Negative*** ***885***	***Positive*** ***315***	***Negative*** ***791***	***Positive*** ***223***	***Negative*** ***682***
*CHE*	*95* (22.41%)*	*116* *(13.11%)*	*82* *(26.03%)*	*135* *(17.07%)*	*59* *(26.46%)*	*104* *(15.25%)*

Source: Prepared by authors based on CONPAS 2018, 2019 and 2020. * The percentage was calculated in relation to the SRQ

These results indicate that, in all years studied, individuals with SRQ + have a higher incidence of CHE compared to those with SRQ-. The percentage of individuals with SRQ + incurring these expenditures increased from 22.41% in 2018 to 26.46% in 2020, highlighting a growing financial burden on those with mental health disorders. These findings underscore the compounded financial vulnerabilities faced by individuals with mental health disorders, particularly in the context of the ongoing armed conflict and the exacerbating effects of the COVID-19 pandemic. Addressing these financial burdens is crucial for promoting mental health and overall well-being in conflict-affected regions.

**Table 5:** CHE according to socioeconomic variables

	2018			2019			2020		
	Catastrophic expenditures (20%)								
	No	Yes	Total	No	Yes	Total	No	Yes	Total
Gender									
Male									
Frequency	505	95	600	409	100	509	330	75	405
Percent	38.58	7	45.84	36.98	9.04	46.02	36.46	8.29	44.75
Female									
Frequency	593	116	709	480	117	597	412	88	500
Percent	45.3	9	54.16	43.4	10.58	53.98	45.52	9.72	55.25
Age group									
18–44									
Frequency	531	67	598	373	83	456	328	35	363
Percent	40.57	5.12	45.68	33.73	7.5	41.23	36.24	3.87	40.11
45–60									
Frequency	354	76	430	311	66	377	254	63	317
Percent	27.04	5.81	32.85	28.12	5.97	34.09	28.07	6.96	35.03
> 60									
Frequency	213	68	281	205	68	273	160	65	225
Percent	16.27	5.19	21.47	18.54	6.15	24.68	17.68	7.18	24.86
HPI									
1									
Frequency	210	53	263	167	58	225	141	46	187
Percent	16.04	4.05	20.09	15.1	5.24	20.34	15.58	5.08	20.66
2									
Frequency	208	55	263	169	51	220	136	39	175
Percent	15.89	4.2	20.09	15.28	4.61	19.89	15.03	4.31	19.34
3									
Frequency	214	46	260	175	46	221	152	29	181
Percent	16.35	3.51	19.86	15.82	4.16	19.98	16.8	3.2	20
4									
Frequency	240	27	267	189	37	226	155	30	185
Percent	18.33	2.06	20.4	17.09	3.35	20.43	17.13	3.31	20.44
5									
Frequency	226	30	256	189	25	214	158	19	177
Percent	17.27	2.29	19.56	17.09	2.26	19.35	17.46	2.1	19.56
SRQ									
Negative									
Frequency	769	116	885	656	135	791	578	104	682
Percent	58.75	8.86	67.61	59.31	12.21	71.52	63.87	11.49	75.36
Positive									
Frequency	329	95	424	233	82	315	164	59	223
Percent	25.13	7.26	32.39	21.07	7.41	28.48	18.12	6.52	24.64
Ethnicity									
Minority									
Frequency	230	52	282	193	51	244	158	33	191
Percent	17.57	3.97	21.54	17.45	4.61	22.06	17.46	3.65	21.1
Majority									
Frequency	868	159	1,027	696	166	862	584	130	714
Percent	66.31	12.15	78.46	62.93	15.01	77.94	64.53	14.36	78.9
Marital status									
Married									
Frequency	219	62	281	206	48	254	154	46	200
Percent	16.73	4.74	21.47	18.63	4.34	22.97	17.02	5.08	22.1
Consensual union									
Frequency	464	72	536	382	99	481	322	62	384
Percent	35.45	5.5	40.95	34.54	8.95	43.49	35.58	6.85	42.43
Separated/Divorced									
Frequency	254	45	299	165	36	201	166	35	201
Percent	19.4	3.44	22.84	14.92	3.25	18.17	18.34	3.87	22.21
Widow(er)									
Frequency	72	22	94	68	19	87	52	12	64
Percent	5.5	1.68	7.18	6.15	1.72	7.87	5.75	1.33	7.07
Single									
Frequency	89	10	99	68	15	83	48	8	56
Percent	6.8	0.76	7.56	6.15	1.36	7	5.3	0.88	6.1
Highest level of education attained									
None									
Frequency	56	23	79	198	76	274	49	12	61
Percent	4.28	1.76	6.04	17.9	6.87	24.77	5.41	1.33	6.74
Preschool/Elementary									
Frequency	442	93	535	331	65	396	292	91	383
Percent	33.77	7.1	40.87	29.93	5.88	35.8	32.27	10.06	42.32
High school									
Frequency	385	54	439	211	48	259	254	38	292
Percent	29.41	4.13	33.54	19.08	4.34	23.42	28.07	4.2	32.27
Technical / Technological / University / Postgraduate									
Frequency	215	41	256	149	28	177	147	22	169
Percent	16.42	3.13	19.56	13.47	2.53	16	16.24	2.43	18.67
Type of work of participant									
Formal									
Frequency	175	29	204	132	32	164	96	13	109
Percent	13.37	2.22	15.58	11.93	2.89	14.83	10.61	1.44	12.04
Informal									
Frequency	820	157	977	690	165	855	591	137	728
Percent	62.64	11.99	74.64	62.39	14.92	77.31	65.3	15.14	80.44
Out of labor force									
Frequency	103	25	128	67	20	87	55	13	68
Percent	7.87	1.91	9.78	6.06	1.81	7.87	6.08	1.44	7.51
Zone of residence									
Rural									
Frequency	421	106	527	371	102	473	303	89	392
Percent	32.16	8.1	40.26	33.54	9.22	42.77	33.48	9.83	43.31
Urban									
Frequency	677	105	782	518	115	633	439	74	513
Percent	51.72	8.02	59.74	46.84	10.4	57.23	48.51	8.18	56.69
Health insurance scheme									
EPS (contributory)									
Frequency	302	48	350	265	58	323	191	38	229
Percent	23.14	3.68	26.82	24.03	5.26	29.28	21.27	4.23	25.5
EPS (Subsidized)									
Frequency	691	138	829	573	146	719	488	113	601
Percent	52.95	10.57	63.52	51.95	13.24	65.19	54.34	12.58	66.93
Exception (For example, armed forces or indigenous people)									
Frequency	43	13	56	19	8	27	28	2	30
Percent	3.3	1	4.29	1.72	0.73	2.45	3.12	0.22	3.34
Unaffiliate									
Frequency	59	11	70	30	4	34	31	7	38
Percent	4.52	0.84	5.36	2.72	0.36	3.08	3.45	0.78	4.23
Hospitalization in the previous 12 months									
No									
Frequency	981	166	1,147	799	172	971	686	136	822
Percent	74.94	12.68	87.62	72.24	15.55	87.79	75.8	15.03	90.83
Yes									
Frequency	117	45	162	90	45	135	56	27	83
Percent	8.94	3.44	12.38	8.14	4.07	12.21	6.19	2.98	9.17
Sick in the previous 12 months									
No									
Frequency	474	51	525	422	71	493	501	72	573
Percent	36.21	3.9	40.11	38.16	6.42	44.58	55.36	7.96	63.31
Yes									
Frequency	624	160	784	467	146	613	241	91	332
Percent	47.67	12.22	59.89	42.22	13.2	55.42	26.63	10.06	36.69
Are there children in the household?									
No									
Frequency	837	174	1,011	662	164	826	534	133	667
Percent	63.94	13.29	77.23	59.86	14.83	74.68	59.01	14.7	73.7
Yes									
Frequency	261	37	298	227	53	280	208	30	238
Percent	19.94	2.83	22.77	20.52	4.79	25.32	22.98	3.31	26.3
Internal displacement									
Yes									
Frequency	440	92	532	355	117	472	319	82	401
Percent	36.27	7.58	43.86	34.8	11.47	46.27	37.89	9.74	47.62
No									
Frequency	575	106	681	463	85	548	370	71	441
Percent	47.4	8.74	56.14	45.39	8.33	53.73	43.94	8.43	52.38
Conflict level									
Not affected									
Frequency	250	44	294	195	54	249	173	41	214
Percent	19.1	3.36	22.46	17.73	4.91	22.64	19.46	4.61	24.07
Villavicencio (Capital city of the region)									
Frequency	271	29	300	206	28	234	151	21	172
Percent	20.7	2.22	22.92	18.73	2.55	21.27	16.99	2.36	19.35
Heavily affected									
Frequency	241	65	306	196	70	266	177	47	224
Percent	18.41	4.97	23.38	17.82	6.36	24.18	19.91	5.29	25.2
Lightly affected									
Frequency	336	73	409	286	65	351	226	53	279
Percent	25.67	5.58	31.25	26	5.91	31.91	25.42	5.96	31.38
Total									
Frequency	1,098	211	1,309	883	217	1,100	727	162	889
Percent	83.88	16.12	100	80.27	19.73	100	81.78	18.22	100

Source: Prepared by authors based on CONPAS 2018, 2019 and 2020

The data analysis shows that there are differences in the incidence of CHE based on individuals’ socioeconomic characteristics. The proportion of women incurring CHE in 2020 is 9.72%, which is higher than that of men, at 8.29%. Likewise, the proportion of individuals experiencing CHE is higher among older individuals, especially those over 60 years old, reaching 7.18% in this age group. Regarding educational level, the proportion of people with CHE is higher among those with lower educational attainment, such as those who only completed primary education (10.06%), compared to those with higher education (2.43%).

The results highlight that individuals with SRQ + face a higher incidence of CHE. Among the 223 individuals with SRQ + in 2020, 59 experienced CHE, representing 26.46%. In contrast, among the 682 individuals with SRQ-, 104 experienced CHE, accounting for 15.24%. Similarly, a higher proportion of individuals who incur CHE work in the informal sector (15.14%) and live in rural areas (9.83%) compared to those with formal employment (1.44%) and those living in urban areas (8.18%). These results suggest a strong relationship between socioeconomic vulnerability, limited access to resources, and CHE, especially among individuals with mental health disorders.

Additionally, throughout the years 2018, 2019, and 2020, consistent trends in the incidence of CHE are observed, although with some variations. In 2018, the total percentage of people who incurred CHE was 16.12%, increasing to 19.73% in 2019, then slightly decreasing to 18.22% in 2020. On the other hand, in terms of mental health, individuals with SRQ + experienced a decrease in the incidence of CHE, from 7.26% in 2018 to 6.52% in 2020.

### Presence of health expenses and catastrophic health expenses

Table 6 presents the results of the mixed-effects logistic regression models, which examine the presence of CHE, defined as an OOP expenditures exceeding 20% of household consumption, as well as OOP expenditures. The analysis covers various socio-economic and demographic variables.

**Table 6:** Determinants of OOP and CHE: results from the Mixed-effects logistic regression models

	Model 1: Catastrophic health expenditures (CHE)			Model 2: Out-of-Pocket health expenditures (OOP)		
	Odds ratio	P>|z|	[95% conf. Interval]	Odds ratio	P>|z|	[95% conf. Interval]
Year (Refence: Year 2018)						
2019	1.40	0.025	(1.04; 1.89)	0.70	0.005	(0.55; 0.9)
2020	1.43	0.018	(1.06; 1.93)	0.50	0.000	(0.39; 0.64)
SRQ (Refence: Negative)						
Positive	1.28	0.071	(0.98; 1.67)	1.44	0.004	(1.12; 1.85)
PQ (Refence: Quintile 5 - richest)						
1	2.34	0.004	(1.3; 4.22)	0.84	0.474	(0.52; 1.35)
2	2.17	0.003	(1.31; 3.61)	0.88	0.555	(0.58; 1.34)
3	1.73	0.022	(1.08; 2.75)	1.01	0.942	(0.7; 1.48)
4	1.27	0.286	(0.82; 1.95)	1.01	0.957	(0.71; 1.43)
Age (Refence: 18–44 years)						
45–60	1.43	0.046	(1.01; 2.05)	1.07	0.636	(0.8; 1.43)
> 60	2.51	0.000	(1.64; 3.83)	1.52	0.024	(1.06; 2.18)
Ethnicity (Refence: Majority)						
Minority	0.87	0.416	(0.62; 1.22)	0.96	0.801	(0.71; 1.3)
Gender (Refence: Male)						
Women	1.27	0.109	(0.95; 1.69)	1.19	0.174	(0.93; 1.53)
Marital Status (Refence: Single)						
Married	1.46	0.220	(0.8; 2.68)	1.74	0.039	(1.03; 2.96)
Consensual Union	1.20	0.526	(0.68; 2.14)	1.61	0.064	(0.97; 2.65)
Divorced	0.86	0.615	(0.47; 1.56)	1.14	0.608	(0.69; 1.9)
Widow/er	0.90	0.785	(0.42; 1.93)	1.34	0.390	(0.68; 2.64)
Education (Refence: Undergraduate)						
None	0.90	0.718	(0.51; 1.59)	0.77	0.299	(0.46; 1.27)
Primary school	0.74	0.186	(0.47; 1.16)	0.79	0.214	(0.54; 1.15)
Secondary school	0.83	0.379	(0.54; 1.26)	0.75	0.095	(0.53; 1.05)
Work (Refence: Formal employee)						
Informal	0.90	0.676	(0.55; 1.47)	0.97	0.898	(0.66; 1.45)
Out of labor force	1.25	0.472	(0.68; 2.27)	1.22	0.460	(0.72; 2.05)
Residence (Refence: Urban)						
Rural	1.02	0.925	(0.72; 1.45)	1.34	0.043	(1.01; 1.79)
Health insurance scheme (Refence: Contributive)						
Subsidized	1.00	0.995	(0.68; 1.47)	0.74	0.071	(0.54; 1.03)
Exception	2.01	0.083	(0.91; 4.42)	1.27	0.401	(0.73; 2.21)
Not affiliated	1.08	0.837	(0.52; 2.27)	1.06	0.841	(0.59; 1.93)
Sick in the previous 12 months (Refence: No)						
Yes	1.97	0.000	(1.52; 2.54)	2.55	0.000	(2.07; 3.15)
Hospitalization in the previous 12 months (Refence: No)						
Yes	2.34	0.000	(1.68; 3.27)	1.69	0.002	(1.22; 2.36)
Children under 6 years old living in the household (Refence: No)						
YES	1.22	0.262	(0.86; 1.74)	1.22	0.182	(0.91; 1.63)
Number of people residing in the household						
Number of people in the household	0.91	0.040	(0.83; 0.99)	1.00	0.905	(0.93; 1.08)
Internal displacement (Refence: Not displaced)						
Yes	1.14	0.386	(0.85; 1.52)	1.31	0.039	(1.01; 1.69)
Conflict level (Refence: No conflict)						
Capital city	0.72	0.169	(0.45; 1.15)	0.62	0.012	(0.42; 0.9)
Heavily affected	1.43	0.090	(0.95; 2.17)	1.34	0.109	(0.94; 1.93)
Lightly affected	1.07	0.705	(0.74; 1.56)	1.13	0.491	(0.8; 1.58)
Constant	0.04	0.000	(0.02; 0.09)	0.67	0.254	(0.34; 1.33)
Wald chi2	147.51			203.59		
Prob > chi2	0.0000			0.0000		

Source: Prepared by authors based on CONPAS 2018, 2019 and 2020

The results of the mixed-effects logistic regression models reveal significant disparities in the odds of incurring both CHE and OOP expenditures, depending on the different determinants. The odds of experiencing CHE increased significantly in 2019 (OR = 1.40, 95% CI 1.04–1.89) and 2020 (OR = 1.43, 95% CI 1.06–1.93) relative to 2018, suggesting a growing health-related financial burden. In contrast, the odds of incurring OOP expenses decreased significantly in 2019 (OR = 0.7, 95% CI 0.55; 0.9) and 2020 (OR = 0.5, 95% CI 0.39; 0.64) compared to 2018.

Over the period, the odds of experiencing CHE were 1.28 (95% CI 0.98; 1.67) times higher for people with an SRQ + compared to those that do not present a mental health disorder, although this result is marginally significant (*p* = 0.071). In contrast, people with SRQ + experienced significantly higher odds (OR = 1.44, 95% CI 1.12; 1.85) of incurring OOP expenses compared to those with SRQ-.

Households in the lower wealth quintiles have significantly higher odds of experiencing CHE compared to those in the wealthiest quintile. Specifically, during these years, the odds ratios were 2.34 (95% CI 1.30; 4.22) for quintile 1, 2.17 (95% CI 1.31; 3.61) for quintile 2, and 1.73 (95% CI 1.08; 2.75) for quintile 3, all significantly higher in comparison with quintile 5, indicating a progressively lower risk of CHE as household wealth increases. Regarding the odds of incurring OOP expenses, there are no significant differences based on household wealth level.

Over the period, the odds of experiencing CHE were higher for the 45–60 age group (OR = 1.43, 95% CI 1.01; 2.05) and for those over 60 years old (OR = 2.51, 95% CI 1.64; 3.83) compared to the younger group aged 18–44 years. Regarding the odds of experiencing OOP according to age, the difference was only significant for those over 60 years old (OR = 1.52, 95% CI 1.06; 2.18) compared to the 18–44 age group. According to the area of residence, the odds of experiencing OOP were higher for people residing in rural areas (OR = 1.34, 95% CI 1.01; 1.79) compared to those in urban areas, while during these years this difference was not significant for the odds of experiencing CHE.

The odds of experiencing CHE were 1.97 (95% CI 1.52; 2.54) and the odds of experiencing OOP were 2.55 (95% CI 2.07; 3.15), higher during the period for those who have fallen sick in the last 12 months compared to those who have not. Additionally, over these years, the odds of experiencing CHE were 2.34 (95% CI 1.68; 3.27) and the odds of experiencing OOP were 1.69 (95% CI 1.22; 2.36), higher for those who have been hospitalized in the last 12 months compared to those who have not. It is important to highlight that while both falling sick and being hospitalized increase the risk of experiencing higher CHE and OOP, falling sick has greater implications on OOP and being hospitalized has greater implications on CHE. This suggests that more severe diseases increase the risk of CHE.

Table 6 also shows that, over the period, for each additional person living in the household, the odds of incurring CHE decrease (OR = 0.91, 95% CI 0.83; 0.99). Additionally, people who have been displaced at some point in their lives have 1.31 (95% CI 1.01; 1.69) times higher odds of experiencing OOP compared to those who have not. People living in municipalities heavily affected by armed conflict have higher odds (OR = 1.43, 95% CI 0.95; 2.17) of experiencing CHE compared to those living in non-conflict municipalities; however, this difference is marginally significant (*p* = 0.090).

### Financial sources used to cover health expenditures

Table 7 presents data on the various financial sources individuals used to cover health expenditures across the years 2018, 2019, and 2020.

**Table 7:** Number of people who use each type of financial source – CONPAS 2018, 2019, 2020

Number of people who use each type of financial source					
Year/ Financial source	Income	Savings	Selling Assets	Borrowing	Total sources
2018 (N: 1309)	626 71.54%	78 8.91%	28 3.2%	143 16.34%	875 100%
2019 (N: 1106)	488 76.72%	29 4.55%	23 3.61%	96 15.09%	636 100%
2020 (N: 905)	306 63.48%	65 13.48%	33 6.84%	78 16.18%	482 100%

Source: Prepared by authors based on CONPAS 2018, 2019 and 2020

The data shows a decreasing trend in the use of income as a source of financing OOP, with a notable drop from 71.54% in 2018 to 63.48% in 2020. The use of savings fluctuated, with a decrease in 2019 but a rise in 2020, surpassing the percentage in 2018. The percentage of individuals selling assets to cover OOP expenses remained relatively low but showed a slight increase in 2020. There was a decrease in the percentage of individuals borrowing money from 2018 to 2019, with a slight further decrease in 2020.

The results highlight shifts in the financial strategies used by individuals to cover health expenditures over the three years. The reliance on income decreased markedly from 2018 to 2020, indicating a possible increased financial strain or reduced income availability due to the economic impacts of the COVID-19 pandemic. Although the use of savings increased in 2020, this may reflect a depletion of other financial resources, pushing individuals to rely more on their savings. The slight increase in the sale of assets and borrowing in 2020 suggests that households are resorting to more desperate measures to cover health costs, highlighting the increased financial vulnerability during the pandemic. These trends underscore the need for robust financial protection mechanisms to support households in managing health expenditures without compromising their financial stability.

### Funding sources and SRQ

Table 8 provides an analysis of the likelihood that individuals with the SRQ + use different financing sources for OOP compared to individuals with SRQ-. The dependent variables analyzed are Income (model 1), Saving (model 2), Selling Assets (model 3), and Borrowing (model 4), which are binary in nature. Each indicates whether a person primarily resorted to a specific source to finance their OOP health expenditures.

Our main variable of interest is SRQ, and we seek to evaluate its impact on different financing strategies. Additionally, year effects control was included in the models to identify possible variations in financing sources over the years. The estimates include various explanatory variables (socioeconomic variables), but in this analysis, we only show the effects of the SRQ and the control variable for the year. The complete logistics regressions with all explanatory variables are detailed in Appendix 8, allowing interested readers to examine the full specification of the models. We clarify that these models have an explanatory purpose only and not a predictive one.

**Table 8:** Logistic regression models that explains the main financial sources for health expenditures, according to years and SRQ

	Dependent variable			
	Model 1: Income	Model 2: Saving	Model 3: Selling Assets	Model 4: Borrowing
SRQ (Base: Negative)				
Positive (dy/dx)	-0.010	0.031	0.010	0.049
Standard error	0.023	0.012	0.009	0.015
*p*-value	0.666	0.010	0.274	0.001
Year (Base: 2018)				
Year 2019 (dy/dx)	-0.027	-0.041	-0.003	-0.016
Standard error	0.025	0.011	0.008	0.015
*p*-value	0.288	0.000	0.723	0.296
Year 2020 (dy/dx)	-0.137	0.019	0.022	-0.017
Standard error	0.025	0.015	0.010	0.015
*p*-value	0.000	0.199	0.032	0.261
Prob > chi2	0.0000	0.0000	0.0000	0.0000

Source: Prepared by authors based on CONPAS 2018, 2019 and 2020

The analysis reveals significant differences in the ways individuals with SRQ + finance their health expenses compared to those with SRQ-. Specifically, individuals with SRQ + are more likely to rely on their savings to cover healthcare costs than their SRQ- counterparts, suggesting that this group may deplete personal financial resources more frequently when addressing healthcare needs. Additionally, those with SRQ + are more likely to resort to borrowing or taking out loans to finance health expenses, underscoring their greater financial vulnerability and the potential for long-term economic consequences. These findings suggest that individuals with mental health disorders face more significant challenges in managing healthcare costs.

## Discussion

The results of this study provide important evidence on the differences in the odds of incurring CHE and OOP expenses, with special emphasis on differences between individuals with a positive tendency to present mental health disorders and those without such conditions, as well as inequalities according to socioeconomic status and other relevant characteristics such as gender, age, ethnicity, among others. In line with previous studies, the findings show that people with mental health disorders are more likely to face financial difficulties due to OOP health expenses [40, 76–80].

In addition, these results highlight the financial vulnerability faced by people with mental health problems and emphasize the need to improve both access and financial coverage for mental health care, as stated in SDG 3 [43, 81]. Ensuring accessible and affordable mental health services, it is crucial that public health policies address the structural barriers that prevent these individuals from receiving timely and quality care [82, 83]. Lack of adequate coverage for mental health services can lead to a cycle of high costs and deteriorating well-being, exacerbating social and economic inequalities [38, 84]. Thus, strengthening financial protection mechanisms and ensuring equitable access for all populations, regardless of their socioeconomic status, would contribute not only to the achievement of SDG 3, but also to the reduction of inequalities (SDG 10) and the promotion of comprehensive wellbeing in vulnerable communities [18, 43, 85, 86].

Although OOP decreased in 2020 compared to 2018, the likelihood of incurring these expenditures remains significantly higher for SRQ + individuals. The reduction in OOPs overall is a positive move toward financial protection and universal health coverage, but the data demonstrates that notable gaps persist among the most vulnerable groups, particularly regarding mental health care. Similarly, people with SRQ + were more likely to incur CHE than people with SRQ-. This is an interesting result, since, in 2020 compared to 2019 and 2018, simultaneously there is a slight reduction in OOP and an increase in CHE.

The mixed-effects logistic regression models provide a more profound understanding of the elements that impact CHE and OOP. The notable rise in the odds of experiencing CHE in 2020, as opposed to 2018, highlights the worsening impact of the pandemic on financial vulnerabilities. The marginal decrease odds of incurring OOP in 2020 may be indicative of the diminished total consumption of healthcare services during the pandemic, which aligns with worldwide observations throughout the pandemic [87, 88], nevertheless, the cost impact continues to be substantial, but this requires further exploration.

Likewise, socioeconomic inequalities continue to be a determining factor in the incidence of CHE [10, 81]. Our results show that individuals with lower incomes face significantly higher odds of incurring CHE compared to higher income groups. However, these inequalities are not reflected in the OOPs, suggesting that individuals with lower incomes may have access to financial protection mechanisms for minor expenses, but not for catastrophic events, which exacerbates the vulnerability of these groups. This raises important implications for public policy, as SDG 10, which focuses on reducing inequalities, has not yet been fully achieved within the health system. It is essential that public health policy addresses mental health from a more cross-cutting perspective that considers both the existence of inequalities and the social determinants of mental health [18, 84].

The findings of this study underscore the importance of addressing social determinants of health, which are fundamental drivers of health inequities [34, 89]. For individuals in conflict-affected regions, these determinants include economic instability, limited healthcare access, and lack of mental health resources, factors that compound existing vulnerabilities and exacerbate financial strain [37, 38]. Addressing health inequities in these regions requires policies that target both direct healthcare interventions and the broader social conditions that influence health. By focusing on social determinants, policymakers can address the root causes of health disparities, which are critical to achieving SDGs related to health and well-being [37].

One study about CHE carried out in Colombia, which use a threshold when OOP is equal to or greater than 20% of their ability to pay, conclude that 9.6% of Colombian households incurred CHE, with differences according to region [45]. Our results suggest that the Meta region has a higher percentage of households incurring catastrophic expenditures than the national average, because, in 2020, 18.22% of the people surveyed in CONPAS presented CHE. This may be related to the strong and historical presence of the armed conflict in the region, which generated lags in hospital infrastructure and difficulties for people to access health services. Additionally, this region still faces significant challenges such as poverty, inequality and unemployment. This further highlights the importance of the socioeconomic determinants in the OOP and CHE, and in this context, the presence of the armed conflict also becomes a relevant determinant and, at the same time, correlated with the living conditions of the population.

The household economic conditions play a crucial role in determining the level of financial stress, as those in lowest wealth quintiles have to face higher levels of CHE. This discovery emphasizes the deep-rooted disparities in the affordability of healthcare, which calls for policies that improve financial security for impoverished households [44, 90]. In addition to the economic status of the household, hospitalization, the presence of an elderly household member, the presence of a family member with a chronic illness or disability are also significant factors affecting CHE [91]. These variables coincide with our results, which also include variables such as mental health status and the impact of armed conflict.

On the other hand, social determinants frequently cluster and interact, for example, a young person who is a victim of an armed conflict or a woman is more likely to live in poverty, which in turn increases their vulnerability to common mental disorders [37, 92]. Additionally, in Colombia, it has been studied those factors such as extended families, households with children or elderly adults, located in rural areas and not covered by the health system, increase CHE [45]. Similarly, factors such as gender, age, and the absence of social security are associated with poverty and mental health disorders, which are in turn determinants of CHE [54, 93, 94]. This highlights a complex intersection between gender, poverty, informal employment, mental health, the impact of armed conflict, and other determinants of CHE.

Furthermore, the results show that the poorest people face higher risks of CHE, and these, in turn, are often individuals with low educational levels, precarious employment, or larger households [45, 60, 91, 92, 95]. Additionally, women have a higher likelihood of incurring CHE, possibly linked to their higher incidence of poverty, informal employment, and other gender inequalities [60, 94, 96]. Individuals with a positive tendency to experience mental health disorders also have a higher likelihood of facing CHE and a higher incidence of poverty [18, 39, 97], while armed conflict exacerbates these conditions by affecting both mental health and economic opportunities [4, 19, 20, 37, 98]. Although the models used in this article do not allow for direct analysis of these interrelations and interdependencies among the determinants of CHE, we emphasize the need to delve deeper into their research to design comprehensive policies that reduce economic and gender inequalities, strengthen social protection, and improve access to mental health services, particularly in conflict-affected communities.

Geographical disparities are evident, with rural residents more likely to have OOP expenses compared to their urban counterparts [99]. This disparity points to the need for improved healthcare infrastructure and financial protection in rural areas. Additionally, individuals who were sick or hospitalized in the previous year had significantly higher odds of incurring CHE, underscoring the high costs associated with severe health events [100, 101].

Households use different financial sources to cover health expenditures, demonstrating their coping techniques. The reduced dependence on earnings and the growing utilization of savings and loans in 2020 suggest an elevated level of financial stress. Individuals who have a SRQ + tendency are more prone to utilizing their resources and taking out loans, which indicates the overall financial challenges they have.

In conclusion, this study highlights the significant financial burdens of health expenditures in conflict-affected regions of Colombia, particularly for individuals with mental health disorders. The findings underscore the need for targeted interventions to reduce financial vulnerabilities and improve access to healthcare. Enhancing financial protection mechanisms and addressing the broader socioeconomic determinants of health are crucial steps towards promoting well-being and economic stability in these regions. As we move forward, integrating mental health into broader development agendas and focusing on reducing health inequities will be essential for achieving sustainable development goals and improving the lives of vulnerable populations [43, 102].

This study presents some limitations that should be considered when interpreting the findings. Firstly, descriptive analysis has an exploratory scope. Secondly, while mixed-effects logistic regression models were used to perform an inferential analysis on the determinants of CHE and OOP expenses, these models do not allow for causal relationships. Additionally, the analysis period is limited to the years 2018, 2019, and 2020, which may not capture broader trends over time. Furthermore, the logistic regression models used to analyze the relationship between SRQ, and the sources of OOP financing were developed with an explanatory, not predictive, approach, given the imbalance in the dependent variable (use of certain financing methods). Finally, the models used do not allow for direct evaluation of the interrelations and interdependencies between the determinants of CHE and OOP, requiring further research that could offer a more comprehensive understanding of the factors at play.

It is also important to consider that we focus on the case of the Meta region. While useful for local health policy, further studies are needed to assess the external validity of our conclusions at the national level in Colombia and in other national settings. Additionally, the SRQ-20 instrument is a screening tool whose scores strongly correlate with the presence and future diagnosis of mental health disorders, yet it does not constitute a clinical diagnosis tool. Despite these limitations, our study is novel in analyzing mental health as a determinant of catastrophic health expenditures and out-of-pocket health spending in contexts affected by armed conflict. This focus contributes to the growing recognition of the intersection between mental health and financial vulnerability in fragile settings, offering valuable insights to guide both health and social policy interventions.

## Electronic supplementary material

Below is the link to the electronic supplementary material.

Supplementary Material 1

## Data Availability

Data will be made available on request.

## Footnotes

1 Appendix 1 contains the Table of descriptive statistics of the CONPAS database for the years 2018, 2019, and 2020.

2 “See Appendix 2 for the attrition analysis.

3 See Appendix 3 for the Cronbach’s alpha of SRQ.

4 See appendix 4 for a more detailed description of the socioeconomic variables.

5 See appendix 5 for a description of the HWI variable construction.

6 See appendix 6 for the statistical tests and model fit criteria (Mixed-effects logistic regressions).

7 See appendix 2 for the attrition analysis.

8 See Appendix 7 for the specification of the logistic regression models.

## Abbreviations

CHE: Catastrophic health expenditures
OOP: Out-of-pocket health expenditures
SRQ-20: Self-report questionnaire
SRQ: Based on the SRQ-20 result, it is concluded that the person either has (SRQ+) or does not have (SRQ-) a positive tendency to present mental health disorders
CMD: Common mental disorders
HWI: Household wealth index
SDG: Sustainable development goals
SGSSS: General system of social security in health
LMIC: Lowe-middle income country
