# Supplementary material for: Mental health and catastrophic health expenditures in conflict-affected regions of Colombia before and during COVID-19: an inequalities perspective
Source: Int J Equity Health. 2025 May 22;24:146. doi: 10.1186/s12939-025-02485-4 (PMC12100806; doi:10.1186/s12939-025-02485-4)
Supplement: Supplementary file 1 — Supplementary Material 1 [file 12939_2025_2485_MOESM1_ESM.docx]

**Appendix**

**Appendix 1: Table of descriptive statistics of the CONPAS database**

|  | 2018 | | 2019 | | 2020 | |
| --- | --- | --- | --- | --- | --- | --- |
|  | N=1309 | | N=1106 | | N=905 | |
|  | Obs. | Percent | Obs. | Percent | Obs. | Percent |
| Gender^⁋^ | | | | | | |
| Men | 600 | 45.84 | 509 | 46.02 | 405 | 44.75 |
| Women | 709 | 54.16 | 597 | 53.98 | 500 | 55.25 |
| Age group | | | | | | |
| 18-44 | 598 | 45.68 | 456 | 41.23 | 363 | 40.11 |
| 45-60 | 430 | 32.85 | 377 | 34.09 | 317 | 35.03 |
| >60 | 281 | 21.47 | 273 | 24.68 | 225 | 24.86 |
| Household poverty Index (HPI) | | | | | | |
| 1 (Poorest) | 263 | 20.09 | 225 | 20.34 | 187 | 20.66 |
| 2 | 263 | 20.09 | 220 | 19.89 | 175 | 19.34 |
| 3 | 260 | 19.86 | 221 | 19.98 | 181 | 20 |
| 4 | 267 | 20.4 | 226 | 20.43 | 185 | 20.44 |
| 5 | 256 | 19.56 | 214 | 19.35 | 177 | 19.56 |
| SRQ | | | | | | |
| SRQ+ | 424 | 32.39 | 315 | 28.48 | 223 | 24.64 |
| SRQ- | 885 | 67.61 | 791 | 71.52 | 682 | 75.36 |
| Work | | | | | | |
| Formal | 204 | 15.58 | 164 | 14.83 | 109 | 12.04 |
| Informal | 977 | 74.64 | 855 | 77.31 | 728 | 80.44 |
| Out of labor force | 128 | 9.78 | 87 | 7.87 | 68 | 7.51 |
| Ethnicity^⁋^ | | | | | | |
| Minority | 282 | 21.54 | 244 | 22.06 | 191 | 21.1 |
| Majority | 1,027 | 78.46 | 862 | 77.94 | 714 | 78.9 |
| Marital Status^⁋^ | | | | | | |
| Married | 281 | 21.47 | 254 | 22.97 | 200 | 22.1 |
| Stable partnership | 536 | 40.95 | 481 | 43.49 | 384 | 42.43 |
| Separated or divorced | 299 | 22.84 | 201 | 18.17 | 201 | 22.21 |
| Widower | 94 | 7.18 | 87 | 7.87 | 64 | 7.07 |
| Single | 99 | 7.56 | 83 | 7.5 | 56 | 6.19 |
| Education Level^⁋^ | | | | | | |
| No formal education | 79 | 6.04 | 274 | 24.77 | 61 | 6.74 |
| Preschool or primary | 535 | 40.87 | 396 | 35.8 | 383 | 42.32 |
| High school | 439 | 33.54 | 259 | 23.42 | 292 | 32.27 |
| Higher education | 256 | 19.56 | 177 | 16 | 169 | 18.67 |
| Area of residence | | | | | | |
| Rural | 527 | 40.26 | 473 | 42.77 | 392 | 43.31 |
| Urban | 782 | 59.74 | 633 | 57.23 | 513 | 56.69 |
| Conflict intensity in the municipality of residence^⁋^ | | | | | | |
|  |  |  | N=1100 |  | N=889 |  |
| Not affected | 294 | 22.46 | 249 | 22.64 | 214 | 24.07 |
| Villavicencio | 300 | 22.92 | 234 | 21.27 | 172 | 19.35 |
| Heavily affected | 306 | 23.38 | 266 | 24.18 | 224 | 25.2 |
| Lightly affected | 409 | 31.25 | 351 | 31.91 | 279 | 31.38 |
| Displaced^⁋^ | | | | | | |
|  | N=1213 |  | N=1020 |  | N=842 |  |
| Yes | 532 | 43.86 | 472 | 46.27 | 401 | 47.62 |
| No | 681 | 56.14 | 548 | 53.73 | 441 | 52.38 |

⁋ Measured during the 2018 CONPAS wave.

**Appendix 2: Attrition analysis.**

Attrition analysis is crucial to ensure the validity of results in longitudinal and panel studies. This analysis helps identify whether the loss of participants over time affects the representativeness and accuracy of the findings. If attrition occurs non-randomly, meaning certain groups of individuals are more likely to drop out of the study, the results may be biased and not reflect the true relationships or effects being investigated. Therefore, conducting an attrition analysis can detect and mitigate these potential biases, ensuring that the study's conclusions are robust and generalizable. In the presented case, by finding no significant differences in health expenditures between the groups included and excluded, it is confirmed that there is no evidence of selective data loss or attrition effect, thus strengthening confidence in the model's results.

| Number of observations (CONPAS 2018) | Health expenditures = Considering the entire sample | Included | *803* |
| --- | --- | --- | --- |
|  |  | Excluded | *506* |
|  |  | Total | *1,309* |
|  | Out of pocket = Excluding those who stated that they had no expenses | Included | *276* |
|  |  | Excluded | *528* |
|  |  | Total | *804* |

| ***Panel A: Test for equality of variances between included and excluded groups*** | | | | |
| --- | --- | --- | --- | --- |
| Null hypothesis (H0) | Alternative hypothesis (H1) | Variable | Test statistic | P-value |
| Ratio of standard deviations (included/excluded) = 1 | Ratio of standard deviations (included/excluded) ≠ 1 | Health expenditures | *F* = 0.6974 | 0.0000 |
|  |  | Health expenditures/ Total household expenditure | *F= 0.9886* | 0.8910 |
|  |  | Out of pocket | *F* = 0.7683 | 0.0141 |
| ***Panel B: Test for difference in means between included and excluded groups***  ***(Two-sample t test with unequal variances)*** | | | | |
| Null hypothesis (H0) | Alternative hypothesis (H1) | Variable | Test statistic | P-value |
| Difference in means (included - excluded) = 0 | Difference in means (included - excluded) ≠ 0 | Health expenditures | *t* = -1.1115 | 0.2666 |
|  |  | Out of pocket | *t* = 0.4412 | 0.3296 |
| ***Panel C: Test for difference in means between included and excluded groups***  ***(Two-sample t test with equal variances)*** | | | | |
| Null hypothesis (H0) | Alternative hypothesis (H1) | Variable | Test statistic | P-value |
| Difference in means (included - excluded) = 0 | Difference in means (included - excluded) ≠ 0 | Health expenditures/Total household expenditure | *t* = -1.5269 | 0.1270 |

**Result of the attrition analysis:**

In the case of mean differences, first, the entire 2018 sample was considered, which corresponds to 1309 observations. Second, a difference in means was made considering only those who reported having some health expenditure (Out of pocket = 1), that is that is, 804 individuals in 2018. Third, a difference in means was made comparing the relationship between health expenditures and total household expenditures (health expenditures/total household expenditures).

The three tests of difference in means between the two groups (group 1: Individuals included in the random effects model; group 2: excluded from the model) show that the means of health expenditures of the people who were included in the model is not different from that of the people who were excluded from the model.

In conclusion, there is no evidence that the data have been selectively lost or there is an attrition effect, therefore, there is no evidence that the model is biased.

**Appendix 3: Cronbach Alpha for SRQ**

First, we clarify the Self-Report Questionnaire (SRQ-20), which consists of 20 questions about general mental health and well-being and was included in the CONPAS survey. It was developed by the World Health Organization (WHO) (1). This instrument is globally accepted, practical, and well-validated for measuring individual tendencies towards mental health disorders, specifically Common Mental Disorders (CMD) such as depression and anxiety (2). In the study, a person is considered to have a positive tendency to experience mental health disorders if they answer 'yes' to 8 or more of the 20 questions in the questionnaire. Therefore, the instrument is used in the same way as in the National Mental Health Survey conducted by the Ministry of Health and Social Protection of Colombia (2015) (3,4).

Secondly, although there is strong evidence demonstrating that it is a universally used, reliable, and well-validated instrument, we conducted a statistical test to evaluate its reliability by calculating Cronbach's alpha. The Cronbach's index, or Cronbach's alpha, is a measure that assesses the internal consistency of a set of items or questions in a questionnaire or test. In simple terms, it indicates how well the items correlate with each other within a scale, reflecting their reliability and internal coherence. (5). The Cronbach's alpha is explained with the following formula:

$$\alpha=\frac{k}{k-a}\left( 1-\frac{\sum s_{i}^{2}}{s_{t}^{2}} \right)$$

Where:

$k$ = Number of test items

$\sum s_{i}^{2}$ = Sum of the variance of each item

$s_{t}^{2}$ = Variance of total test scores

A high value of Cronbach's alpha (usually greater than 0.8) indicates that the items have a high correlation between them and, therefore, the scale is reliable. (5).

We calculated the Cronbach's alpha for each year of analysis taking into account the 20 variables of the questionnaire and all the individuals surveyed, and the result is as follows:

| **Year** | **Cronbach Alpha for SRQ** |
| --- | --- |
| 2018 | 0.8722 |
| 2019 | 0.8979 |
| 2020 | 0.9055 |

This value indicates that the internal reliability of the scale is very good. This suggests that the questions on the scale have high internal consistency, i.e., they consistently measure the same construct (mental health disorders).

**Appendix 4: Description of the Explanatory Socioeconomic Variables**

| **Variable** | **Description** |
| --- | --- |
| Household Wealth Index – HWI | Measures socio-economic status by capturing information about access to various assets. The 5th quintile representing the wealthiest individuals |
| Age group | Three age groups were considered: 18 to 44 years old, 45 to 60 years old, and over 60 years old. |
| Ethnicity | Participants were initially asked to self-identify according to their culture, community, or physical traits within the following categories: Indigenous, Gitano(a)/Rom, Raizal from the archipelago, Palenquero(a) from San Basilio, Black/Mulatto/Afro-Colombian/Afro-descendant, White, Mestizo, or None of the above. To simplify the analysis and improve statistical estimates, we recategorized the ethnicity variable into two groups: majority and minority. According to the CONPAS survey, 42.9% of respondents identified as Mestizo and 35.5% as White, together representing 78.4% of the population (see Appendix 1: Table of descriptive statistics of the CONPAS database). Based on this distribution, we classified as ethnic minorities those who identified as Indigenous, Gitano(a)/Rom, Raizal, Palenquero(a), Black/Mulatto/Afro-Colombian/Afro-descendant, or None of the above. |
| Gender | Binary variable that considers male or female. |
| Marital status | Includes the categories of married, consensual union, separated/divorced, widowed, or single. |
| Educational level | Considers the highest level of education attained among the categories “None”, “Preschool/Elementary”, “High school”, “Technical/Technological, University, and Postgraduate |
| Employment status | Includes the categories of formal employee (makes contributions to the health and social security system), informal employee (includes all paid work that is not regulated by legal or regulatory frameworks), and out of labor force, which includes all individuals who do not work. |
| Area of residence | Whether the person resides in a rural area or an urban area. |
| Health insurance scheme | According to the description given in the introduction about the SGSSS, there are different health regimes in Colombia. The categories considered are contributory, subsidized, exception, and non-affiliated. |
| Health status | Consider whether the person has been sick and/or hospitalized in the last 12 months. |
| Household size | Number of household members |
| Children under 6 years old | Consider whether there are children under 6 years old in the household. |
| Displacement due to armed conflict | Consider whether the person has ever been displaced in their lifetime due to armed conflict. |
| Level of impact of the conflict in the area | Measures the incidence of conflict in the place of residence, with the categories being no conflict, regional capital city, heavily affected by conflict, and lightly affected by conflict. This classification is defined according to the description of the CONPAS survey included in the Data Source section of the manuscript. |

**Appendix 5: Household Poverty Index (HPI) construction.**

We used the Household Poverty Index (HPI) (6), which measures socio-economic status by capturing information about access to various assets. The HPI is defined by the following equation:

$$HPI=\alpha_{1}\left( \frac{X_{1}-\bar{X_{1}}}{S_{1}} \right)+\alpha_{1}\left( \frac{X_{2}-\bar{X_{2}}}{S_{2}} \right)+\ldots\alpha_{k}\left( \frac{X_{k}-\bar{X_{k}}}{S_{k}} \right)$$

where $X_{k}$ represents a variable that measures access to a specific household asset related to wealth (e.g., home appliances), $\bar{X_{k}}$ is the mean of this variable, $S_{k}$ is its standard deviation, and $\alpha_{k}$ is a specific weight for the variable obtained through Principal Components Analysis (PCA), using the first component of the PCA as an estimator. The index is constructed through the weighted summation of *k* variables that measure access to different household assets.

**Appendix 6: Statistical tests and model fit criteria for the mixed effects logistic regression models**

**Intraclass correlation coefficient (ICC)**

| **Model** | **Level** | **ICC** | **Std. err.** | **[95% conf. interval]** | |
| --- | --- | --- | --- | --- | --- |
| 1. (Dep Var. CHE) | id | 0.188 | 0.045 | 0.114 | 0.292 |
| 2. (Dep Var. OOP) | id | 0.210 | 0.035 | 0.149 | 0.288 |

The results of ICC indicated the presence of unobserved heterogeneity across individuals, justifying the inclusion of random effects to account for this variation. A mixed-effects model could be appropriate. (7).

**Test de Hausman:**

Null hypothesis (H0): There is no correlation between the individual unobserved effects ($\alpha_{i}$) and the explanatory variables $(x_{it})$. In this case, the random effects model is consistent and efficient.

Alternative hypothesis (H1): There is a correlation between the individual unobserved effects ($\alpha_{i}$) and the explanatory variables $(x_{it})$. In this case, the random effects model is inconsistent, and the fixed effects model should be used.

| **Dependent variable of Model (Considering the same explanatory variables in all models)** | **Chi2** | **Prob Chi2** |
| --- | --- | --- |
| OOP: Out of pocket. The person has a health expenditure or not | 31.55 | 0.340 |
| Catastrophic expenditures: Considering that the health expenditures exceeded 20% of household consumption | 29.89 | 0.229 |

Conclusion: The null hypothesis that, there is no correlation between the individual unobserved effects ($\alpha_{i}$) and the explanatory variables $(x_{it})$, cannot be rejected. (8,9).

**Akaike Information Criterion (AIC), Bayesian Information Criterion (BIC) and Likelihood-ratio test.**

| **Model** | **AIC** | **BIC** | **Likelihood-ratio test** |
| --- | --- | --- | --- |
| Dependent Variable: OOP | | | Prob > chi2 |
| Mixed effects | 3012.006 | 3208.380 | 0.0000 |
| Random effects | 3012.006 | 3208.379 |  |
| Dependent Variable: CHE | | | Prob > chi2 |
| Mixed effects | 2182.046 | 2366.869 | 0.0240 |
| Random effects | 2178.600 | 2374.974 |  |

The Akaike Information Criterion (AIC) and Bayesian Information Criterion (BIC) values for the mixed-effects and random-effects logistic regression models were comparable.

Additionally, we conducted a Likelihood Ratio Test between the reduced mixed effects logistic regression models (only with the dependent variable and random effects) and the full model (with all explanatory variables included), aiming to evaluate whether the inclusion of independent variables significantly improves the model fit. The results are as follows:

| **Model** | **AIC** | **BIC** | **Likelihood-ratio test** |
| --- | --- | --- | --- |
| **Dependent Variable: OOP** | | | **Prob > chi2** |
| **Mixed effects (reduced)** | 3475.500 | 3487.223 | 0.0000 |
| **Mixed effects (full)** | 3012.006 | 3208.380 |  |
| **Dependent Variable: CHE** | | | **Prob > chi2** |
| **Mixed effects (reduced)** | 2456.301 | 2468.024 | 0.0000 |
| **Mixed effects (full)** | 2182.046 | 2366.869 |  |

Considering the Likelihood-ratio tests results, the null hypothesis that the simpler (restricted) model is as good as the more complex (less restricted) model in explaining the variability of the data is rejected. Therefore, we consider the mixed-effects logistic regression models to be the most appropriate.

**Appendix 7: Logistic regression models specification - validation**

|  |  | **Model 1: Income** | **Model 2: Saving** | **Model 3: Selling Assets** | **Model 4: Borrowing** |
| --- | --- | --- | --- | --- | --- |
| **Hosmer–Lemeshow** | Chi2(8) | 11.07 | 3.73 | 6.05 | 7.53 |
|  | Prob > chi2 | 0.1978 | 0.8803 | 0.6411 | 0.4811 |
| **Area under ROC curve** | | 0.6447 | 0.7237 | 0.7968 | 0.7244 |

The Hosmer-Lemeshow Test is a widely used statistical test to assess the goodness-of-fit of logistic regression models. The null hypothesis is that the model fits well to the data (no significant differences between the observed and predicted proportions). (10). As shown in the results table, all the Prob > chi2 values are greater than 0.05, meaning there is not enough evidence to reject the null hypothesis in any of the models. In other words, the evidence suggests that the model calibration is adequate.

The Receiver Operating Characteristic Curve (ROC curve) is a tool used to evaluate the performance of a logistic regression model. This curve illustrates the model's ability to distinguish between two classes (e.g., 0 and 1) by analyzing the relationship between the true positive rate and the false positive rate for different probability thresholds. A value of 1 indicates that the model is perfect. (10). In our case, the ROC for Model 1 is between 0.6 and 0.7, and for Models 2, 3, and 4, it is between 0.7 and 0.8, indicating an acceptable discrimination.

However, it is important to note that the models were developed with an explanatory, not predictive, approach, given the imbalance in the dependent variable (use of certain financing methods). This approach seeks to identify and understand the significant relationships between variables, rather than maximizing predictive accuracy.

**Appendix 8: Logistic regression models that explains the main financial sources for health expenditures, according to years and SRQ (Supplement to Table 8 of the manuscript)**

|  | Model 1: Income | Model 2: Saving | Model 3: Selling Assets | Model 4: Borrowing |
| --- | --- | --- | --- | --- |
|  | b/se/p^⁋^ | b/se/p | b/se/p | b/se/p |
|  | | | | |
| SRQ (Base: Negative) | | | | |
| Positive | -0.010 | 0.031 | 0.010 | 0.049 |
|  | 0.023 | 0.012 | 0.009 | 0.015 |
|  | 0.666 | 0.010 | 0.274 | 0.001 |
|  | | | | |
| Year (Base: 2018) | | | | |
| Year 2019 | -0.027 | -0.041 | -0.003 | -0.016 |
|  | 0.025 | 0.011 | 0.008 | 0.015 |
|  | 0.288 | 0.000 | 0.723 | 0.296 |
| Year 2020 | -0.137 | 0.019 | 0.022 | -0.017 |
|  | 0.025 | 0.015 | 0.010 | 0.015 |
|  | 0.000 | 0.199 | 0.032 | 0.261 |
|  | | | | |
| Household Poverty Index (Base: Quintile 5 - richest) | | | | |
| Quintile 1 | -0.035 | -0.037 | 0.023 | -0.002 |
|  | 0.043 | 0.024 | 0.020 | 0.025 |
|  | 0.418 | 0.121 | 0.240 | 0.934 |
| Quintile 2 | -0.016 | -0.045 | -0.002 | 0.039 |
|  | 0.039 | 0.023 | 0.015 | 0.023 |
|  | 0.687 | 0.044 | 0.882 | 0.086 |
| Quintile 3 | 0.016 | -0.034 | -0.007 | 0.007 |
|  | 0.036 | 0.022 | 0.014 | 0.021 |
|  | 0.663 | 0.113 | 0.621 | 0.744 |
| Quintile 4 | 0.004 | -0.019 | -0.006 | 0.018 |
|  | 0.033 | 0.022 | 0.015 | 0.020 |
|  | 0.899 | 0.384 | 0.662 | 0.392 |
|  | | | | |
| Age (Base: 18-44 years) | | | | |
| 45-60 | 0.011 | -0.011 | 0.011 | 0.002 |
|  | 0.025 | 0.011 | 0.007 | 0.015 |
|  | 0.669 | 0.324 | 0.135 | 0.909 |
| >60 | 0.016 | 0.017 | 0.034 | 0.065 |
|  | 0.033 | 0.016 | 0.012 | 0.024 |
|  | 0.622 | 0.289 | 0.006 | 0.006 |
|  | | | | |
| Ethnicity (Base: Majority) | | | | |
| Minority | -0.001 | -0.004 | -0.008 | 0.014 |
|  | 0.025 | 0.011 | 0.009 | 0.016 |
|  | 0.954 | 0.702 | 0.374 | 0.382 |
|  | | | | |
| Gender (Base: Male) | |  |  |  |
| Female | -0.005 | 0.016 | 0.023 | 0.007 |
|  | 0.022 | 0.010 | 0.008 | 0.013 |
|  | 0.825 | 0.109 | 0.008 | 0.607 |
|  | | | | |
| Marital Status (Base: single) | | | | |
| Married | 0.095 | 0.065 | 0.000 | -0.023 |
|  | 0.047 | 0.016 | . | 0.030 |
|  | 0.045 | 0.000 | . | 0.438 |
| Consensual Union | 0.067 | 0.039 | 0.000 | -0.012 |
|  | 0.045 | 0.014 | . | 0.029 |
|  | 0.137 | 0.004 | . | 0.678 |
| Divorced | 0.011 | 0.036 | 0.000 | 0.003 |
|  | 0.047 | 0.015 | . | 0.031 |
|  | 0.825 | 0.016 | . | 0.915 |
| Widow/er | 0.051 | 0.025 | 0.000 | -0.006 |
|  | 0.060 | 0.018 | . | 0.037 |
|  | 0.396 | 0.174 | . | 0.876 |
|  | | | | |
| Education (Base: University/Technical/Technological Education.) | | | | |
| None | -0.072 | 0.042 | 0.015 | -0.043 |
|  | 0.045 | 0.030 | 0.019 | 0.028 |
|  | 0.112 | 0.160 | 0.413 | 0.114 |
| Primary school | -0.002 | -0.010 | -0.002 | -0.043 |
|  | 0.035 | 0.017 | 0.014 | 0.022 |
|  | 0.964 | 0.542 | 0.909 | 0.054 |
| Secondary school | -0.038 | 0.001 | 0.014 | -0.017 |
|  | 0.032 | 0.016 | 0.015 | 0.022 |
|  | 0.237 | 0.959 | 0.355 | 0.430 |
|  | | | | |
| Work (Base: Formal job) | | | | |
| Informal | 0.010 | -0.018 | 0.023* | 0.016 |
|  | 0.037 | 0.022 | 0.010 | 0.020 |
|  | 0.795 | 0.401 | 0.016 | 0.416 |
| Out of labor force | 0.020 | -0.034 | 0.034 | 0.049 |
|  | 0.048 | 0.025 | 0.021 | 0.028 |
|  | 0.682 | 0.163 | 0.100 | 0.079 |
|  | | | | |
| Residence (Base: Rural) | | | | |
| Urbano | 0.035 | 0.009 | 0.011 | -0.002 |
|  | 0.026 | 0.011 | 0.009 | 0.016 |
|  | 0.174 | 0.418 | 0.245 | 0.918 |
|  | | | | |
| Health insurance scheme (Base: contributive) | | | | |
| Subsidized | -0.093 | 0.019 | 0.000 | 0.012 |
|  | 0.030 | 0.013 | 0.012 | 0.017 |
|  | 0.002 | 0.132 | 0.991 | 0.503 |
| Excepcion | 0.075 | -0.022 | 0.000 | 0.009 |
|  | 0.056 | 0.017 | . | 0.036 |
|  | 0.180 | 0.210 | . | 0.804 |
| Not affiliated | -0.012 | -0.013 | 0.030 | 0.017 |
|  | 0.061 | 0.023 | 0.028 | 0.040 |
|  | 0.846 | 0.553 | 0.284 | 0.678 |
|  | | | | |
| Sick in the previous 12 months (Base: No) | | | | |
| YES | 0.132 | 0.020 | 0.021 | 0.048 |
|  | 0.021 | 0.010 | 0.008 | 0.013 |
|  | 0.000 | 0.052 | 0.008 | 0.000 |
|  | | | | |
| Hospitalization in the previous 12 months (Base: No) | | | | |
| YES | -0.001 | 0.031 | -0.007 | 0.077 |
|  | 0.031 | 0.017 | 0.010 | 0.023 |
|  | 0.970 | 0.063 | 0.518 | 0.001 |
|  | | | | |
| Children under 6 years old (Base: No) | | | | |
| Yes | 0.036 | -0.005 | -0.010 | 0.019 |
|  | 0.026 | 0.012 | 0.009 | 0.017 |
|  | 0.170 | 0.687 | 0.240 | 0.277 |
|  | | | | |
| Household size | 0.002 | -0.005 | 0.005* | 0.003 |
|  | 0.007 | 0.003 | 0.002 | 0.004 |
|  | 0.821 | 0.131 | 0.033 | 0.437 |
|  | | | | |
| Internal displacement (Base: It has not been displaced) | | | | |
| Yes | 0.017 | 0.007 | 0.002 | 0.053*** |
|  | 0.022 | 0.010 | 0.009 | 0.013 |
|  | 0.436 | 0.481 | 0.822 | 0.000 |
|  |  |  |  |  |
| Conflict level (Base: No conflict) | | | | |
| Capital city | -0.094** | 0.003 | -0.010 | 0.006 |
|  | 0.032 | 0.014 | 0.011 | 0.021 |
|  | 0.003 | 0.832 | 0.361 | 0.791 |
| Heavily affected | 0.047 | 0.020 | 0.011 | -0.005 |
|  | 0.031 | 0.014 | 0.013 | 0.019 |
|  | 0.128 | 0.158 | 0.399 | 0.795 |
| Lightly affected | 0.017 | 0.010 | 0.006 | 0.006 |
|  | 0.028 | 0.012 | 0.010 | 0.017 |
|  | 0.548 | 0.426 | 0.575 | 0.725 |
|  | | | | |
| Number of obs | 2382 | 2382 | 2164 | 2382 |
| Prob > chi2 | 0.0000 | 0.0000 | 0.0000 | 0.0000 |

**References:**

1. Beusenberg M, Orley JH, Organization WH. A User’s guide to the self reporting questionnaire (SRQ. World Health Organization; 1994.

2. Patel V, Araya R, Chowdhary N, King M, Kirkwood B, Nayak S, et al. Detecting common mental disorders in primary care in India: a comparison of five screening questionnaires. Psychol Med. 2008;38(2):221.

3. Ministerio de Salud y Protección Social. Encuesta Nacional de Salud Mental . 2015.

4. Rodriguez V, Moreno S, Camacho J, Gómez-Restrepo C, de Santacruz C, Rodriguez MN, et al. Diseño e implementación de los instrumentos de recolección de la Encuesta Nacional de Salud Mental Colombia 2015. Rev Colomb Psiquiatr [Internet]. 2016;45:9–18. Available from: https://www.sciencedirect.com/science/article/pii/S0034745016301093

5. Medrano L, Pérez E. Manual de psicometría y evaluación psicológica. 2019;

6. Filmer D, Pritchett LH. Estimating wealth effects without expenditure data—or tears: an application to educational enrollments in states of India. Demography. 2001;38(1):115–32.

7. Twisk JWR. Applied Mixed Model Analysis: A Practical Guide [Internet]. 2nd ed. Practical Guides to Biostatistics and Epidemiology. Cambridge: Cambridge University Press; 2019. Available from: https://www.cambridge.org/core/product/16BB3849827F848579608B8C788A51F8

8. Wooldridge JM. Introducción a la econometría. Un enfoque moderno: un enfoque moderno. Editorial Paraninfo; 2006.

9. Verbeek M. A Guide to Modern Econometrics. 2004;

10. Hosmer Jr DW, Lemeshow S, Sturdivant RX. Applied logistic regression. John Wiley & Sons; 2013.
